# Angiotensin-converting enzyme 2 activator diminazene aceturate prevents lipopolysaccharide-induced inflammation by inhibiting MAPK and NF-κB pathways in human retinal pigment epithelium

**DOI:** 10.1186/s12974-016-0489-7

**Published:** 2016-02-09

**Authors:** Lifei Tao, Yiguo Qiu, Xinyu Fu, Ru Lin, Chunyan Lei, Jiaming Wang, Bo Lei

**Affiliations:** Department of Ophthalmology, The First Affiliated Hospital of Chongqing Medical University, Chongqing Key Laboratory of Ophthalmology, Chongqing Eye Institute, 1 You Yi Road, Yu Zhong District, Chongqing, 400016 China

**Keywords:** Diminazene aceturate, Angiotensin-converting enzyme 2, Inflammation, MAPK, NF-κB

## Abstract

**Background:**

Retinal inflammation is a devastating pathological process in ocular diseases. Functional impairment of retinal pigment epithelium (RPE) is associated with inflammatory retinal diseases. Enhancing the protective axis namely ACE2/Ang-(1-7)/Mas by activation of ACE2 presents anti-inflammatory properties. We investigated whether diminazene aceturate (DIZE), an angiotensin-converting enzyme 2 (ACE2) activator, prevented lipopolysaccharide (LPS)-induced inflammatory response by activating the protective axis and whether the effect was mediated by inhibiting the mitogen-activated protein kinase (MAPK) and the nuclear factor-κB (NF-κB) pathways.

**Methods:**

Cell counting kit-8 (CCK-8) assay and real-time PCR were used to determine the optimum concentration and incubation time of DIZE. ARPE-19 cells and primary cultured human retinal pigment epithelia (hRPE) were incubated with or without 10 μg/mL DIZE for 6 h before stimulated with 5 μg/mL LPS for 24 h. The mRNA expression of inflammatory cytokines, AT1R, and AT2R was analyzed. The protein level of inflammatory cytokines, Ang II, and Ang-(1-7) was detected. Phosphorylation of p38 MAPK, extracellular signal-regulated kinase (ERK)1/2, c-Jun N-terminal kinase (JNK) and phosphorylated transcription inhibition factor-κB-α (p-IκB-α) were measured. Inhibitors of MAPKs and NF-κB were added to verify the involvement of these pathways. A small interfering RNA (siRNA) targeted to ACE2 and a selective Ang-(1-7) antagonist A779 was used to confirm the role of ACE2 and the involvement of ACE2/Ang-(1-7)/Mas axis.

**Results:**

DIZE remarkably increased the expression of ACE2 and inhibited the expression of IL-6, IL-8, and MCP-1 at both mRNA and protein levels in both RPE cell lines stimulated with LPS. Inhibitors of p38, ERK1/2, JNK, and NF-κB significantly decreased LPS-induced overproduction of IL-6, IL-8, and MCP-1. DIZE reduced the expression of Ang II and AT1R, whereas increased Ang-(1-7). Furthermore, DIZE downregulated the phosphorylation of p38MAPK, ERK1/2, JNK, and the activation of NF-κB upon stimulation with LPS. Downregulating ACE2 and pre-treatment with A779 abrogated the effects of DIZE on production of cytokines, the expression of Ang II, Ang-(1-7), AT1R, phosphorylation of MAPKs and activation of NF-κB.

**Conclusions:**

DIZE inhibits LPS-induced inflammatory response by activating ACE2/Ang-(1-7)/Mas axis in human RPE cells. The protective effect is mediated by inhibiting the p38MAPK, ERK1/2, JNK, and NF-κB pathways.

## Background

The renin-angiotensin system (RAS) is a complex hormone system and classically known as a regulator of blood pressure. It is also recognized recently as a pro-inflammatory mediator [1]. In addition to systemic RAS, tissue intrinsic RAS has been identified in various tissues including the retina. The malfunction of tissue intrinsic RAS is involved in the pathogenesis of vascular [2], pulmonary [3] and ocular diseases [4]. Angiotensin II (Ang II) is a vital molecular regulator of RAS. It is generated through sequential enzymatic catalysis by angiotensin-converting enzyme (ACE) from angiotensinogen. Ang II has two receptors, angiotensin II type 1 receptor (AT1R) and AT2R. It has been proved that Ang II plays the deleterious roles through its receptor AT1R. Several studies demonstrated that the harmful axis of RAS, i.e., the ACE/Ang II/AT1R axis was involved in several retinal diseases [4, 5]. Therapeutic intervention targeted to this axis showed remarkable protective effects. AT1R blocker (ARB) suppressed retinal neural dysfunction and downregulated the expression of pro-inflammatory cytokines in animal models of acute retinal inflammation [6]. In addition, oral administration of ARB prevented the progression of diabetic retinopathy by attenuating infiltration of leukocytes in the retina and delays the development of diabetic retinopathy [7].

Angiotensin-converting enzyme 2 (ACE2), a newly found component of RAS, is an enzymatic homologue of ACE. ACE2 decreases the generation of Ang II by catalyzing the conversion of Ang II to angiotensin-(1-7) [Ang-(1-7)], which inhibits the vasoconstrictive, inflammatory, and oxidation stress effects of Ang II [8, 9]. Studies show that the ACE2/Ang-(1-7)/Mas axis is the protective axis of RAS [9]. This axis presents a broad range of beneficial effects including improving pathological conditions such as inflammation and fibrosis [10]. In fact, recombinant ACE2 effectively decreases the cell apoptosis rate and inflammatory cytokines protein levels on LPS-induced pulmonary microvascular endothelial cells (PMVECs) by inhibiting c-Jun N-terminal kinase (JNK) and nuclear factor-κB (NF-κB) pathways [11]. Existing evidence considers that increased expression of ACE2 and Ang-(1-7) via adeno-associated virus (AAV)-mediated gene delivery in the retina diminishes diabetes-induced retinal inflammation and vascular pathology [12]. These studies propose an idea that enhancing the ACE2/Ang-(1-7)/Mas axis may be a promising approach for retinal inflammatory diseases.

Recently studies show that diminazene aceturate (DIZE), a classical drug for trypanosomiasis and babesiosis, is an activator of ACE2 [13]. It is capable to increase the enzymatic activity of ACE2, thus activates the protective axis of RAS: ACE2/Ang-(1-7)/Mas [14] and exerts anti-inflammatory role in pathological conditions. It has been suggested that DIZE reduces serum levels of pro-inflammatory cytokines by directly altering the production of these cytokines in macrophages [15], and exerts anti-inflammation effects under many inflammatory pathological processes including myocardia ischemia [13], pulmonary hypertension [16], and endotoxin-induced uveitis (EIU) [14, 17]. However, how DIZE exerts its anti-inflammatory effect as an ACE2 activator remains unclear.

As a part of the central nervous system (CNS), the retina plays an essential role in the formation of vision. The retinal pigment epithelium (RPE) regulates the transport of nutrients and waste products from the retina, and contributes to outer segment renewal by ingesting and degrading the spent debris of photoreceptor outer segments [18]. Thus, RPE plays crucial roles in maintaining normal retinal function [19]. Dysfunction of RPE cells is involved in many inflammatory blind-causing ocular diseases, including age-related macular disease (AMD) [20]. Oxidative stress, lipofuscin accumulation in RPE cells are well-known pathological features of AMD [21]. In addition, inflammation of RPE exacerbates AMD because infiltrating macrophages promote choroidal neovascularization (CNV) [22]. We used primary cultured human RPE isolated from the donor eyes and a human RPE-derived cell line ARPE-19, which has similar structural and functional characteristics of RPE [23] in this study.

Lipopolysaccharide (LPS) induces acute inflammatory responses of RPE in many ocular pathological conditions including bacterial endophthalmitis and uveitis [24, 25]. Interleukin (IL)-6, IL-8, and monocyte chemoattractant protein-1 (MCP-1) are representative cytokines that initiate ocular inflammation. Inflammatory response mediated by LPS stimulation results in secretion of a series of cytokines through IL-6 and IL-8 autocrine signaling in ARPE-19 cells [25]. IL-6 plays a critical role in many intraocular inflammatory diseases [26, 27]. MCP-1 and IL-8 are thought to be the major mediator for neutrophil and monocyte infiltration in retinal inflammatory diseases [28]. It has been revealed that IL-6 and IL-8 play an important role in inflammation associated with the development of AMD [29]. Moreover, MCP-1 contributes to inflammatory disorder and the formation of CNV [30]. IL-6, IL-8, and MCP-1 levels are significantly higher in aqueous humor of patients with exudative AMD [31].

Studies have demonstrated that LPS-activated NF-κB and mitogen-activated protein kinase (MAPK) cascades upon recognition by toll-like receptor 4 on the cellular surface and led to the release of pro-inflammatory cytokines [32]. In addition, DIZE reduces LPS-induced production of inflammatory cytokines by downregulating phosphorylation of MAPK and activation of NF-κB in macrophages [33]. We hypothesize that DIZE attenuates LPS-induced inflammatory response by activating the ACE2/Ang-(1-7)/Mas axis, and the protective effect is mediated by inhibiting the MAPK and NF-κB pathways.

## Methods

### Cell culture

Human primary retinal pigment epithelia (hRPE) were obtained from donors of the Chongqing Eye Bank. The hRPE were obtained using a previously described protocol [34]. Briefly, the cornea, lens and vitreous were removed. Eye cup was rinsed with Ca^2+^ and Mg^2+^ Dulbecco’s PBS allowing prompt separation of the neural retina from the RPE and detachment of the choroid from the sclera. The RPE cells adhering to Bruch’s membrane on the choroidal sheets were washed with Hank’s balanced salt solution (HBSS; GE Healthcare Life Sciences, Waltham, MA, USA) and treated with 0.05 % trypsin and 0.02 % EDTA. The cells were suspended in complete Dulbecco’s modified Eagle’s medium F-12 nutrient mixture (DMEM/F-12; Invitrogen, Carlsbad, CA) containing 10 % fetal bovine serum, 100 U/mL penicillin (Beyotime, Shanghai, China) and 100 μg/mL streptomycin (Beyotime), then transferred to 25-cm^2^ flasks. The cells were incubated in a humidified incubator at 37 °C in 5 % CO_2_. The homogeneity of cultured RPE cells was confirmed by positive immunostaining with RPE65, a specific marker of RPE cells. The cells used were from passage 3~4.

Human RPE cell line ARPE-19 was purchased from the American Type Culture Collection (ATCC; Manassas, CA). Cells were cultured in DMEM/F12 (Dulbecco’s modified Eagle’s medium: nutrient mixture F12, 1:1, Invitrogen, Carlsbad, CA) medium containing 10 % fetal bovine serum (FBS; Invitrogen), 100 U/ml penicillin, and 100 μg/mL streptomycin. The cells were cultured at 37 °C in humidified 5 % CO_2_ condition and detached with 0.25 % trypsin-EDTA (Invitrogen) solution, diluted 1:3 to 1:4, and plated for subculture. The ARPE-19 cells used in the experiments were between passage 20 and 25.

### Immunofluorescence staining

Isolated hPRE and ARPE-19 cells were seeded onto a glass coverslips and incubated at 37 °C and 5 % CO_2_ until 80 % confluent. Cells were fixed with 4 % paraformaldehyde for 15 min at room temperature, followed by three times wash with PBS. Fixed cells were incubated with 0.1 % triton X-100 for 4 min and then blocked in PBS containing 10 % goat serum for 30 min. Cells were incubated with anti-RPE65 antibody (Abcam, Cambridge, MA, USA) with the concentration of 1:100 overnight at 4 °C. Cells were washed three times in PBS and incubated with goat anti-mouse IgG secondary antibody (DyLight 594; Abbkine, CA, USA) for 30 min at room temperature, followed by 2 min of nuclear staining with DAPI. All fluorescent images were taken with a fluorescence microscope (Model DM6000; Leica, Wetzlar, Germany).

### Cell viability assay

The effect of DIZE (Santa Cruz Biotechnology, Inc., Santa Cruz, CA) on the viability of ARPE-19 cells were determined using the cell counting kit-8 (CCK-8, Sigma-Aldrich, St. Louis, MO) according to the manufacturer’s instructions. ARPE-19 cells were plated in 96-well plates at a density of 1 * 10^4^ cells per well. After 2 days’ incubation, the medium was withdrawn. The cells were washed with PBS and starved with serum-free medium for 24 h and followed by pre-incubation with different concentrations (0.01, 0.1, 1, 10, 100 μg/mL) of DIZE for 3, 6, and 12 h, then stimulated with 5 μg/mL LPS (Sigma-Aldrich) for 24 h. Afterwards, 10 μl WST-8 was added to each well, and incubated for 4 h. The optical density was read at 450 nm using a multifunction microplate reader (Molecular Devices, Sunnyvale, CA). Cells cultured without DIZE were used as a control.

### Experiment protocols

The hRPE and ARPE-19 cells were plated into 24-well plates at a density of 2 * 10^5^ cells per well and cultured in DMEM/F12 medium containing 10 % FBS for 2 days to become confluent. Before treatment, cells were starved for 24 h in serum-free medium. RPE cells were pre-incubated in serum-free medium with or without 10 μg/mL of DIZE for 6 h. Then, 5 μg/mL LPS was added to the medium. Cells were cultured for 24 h, and the supernatants were collected for ELISA assay. The cells were subsequently harvested for mRNA and protein assays. The DIZE-only group was treated the same way as in DIZE + LPS group, except for adding LPS. The assay samples were collected at the same time as in the DIZE + LPS group.

To confirm the involvement of ACE2/Ang-(1-7)/Mas axis, hRPE and ARPE-19 cells were seeded into 24-well plates to become confluent. Then serum-free starved for 24 h and pre-incubated for 30 min with 10 μM of the Ang-(1-7) antagonist, A779 (Santa Cruz Biotechnology), followed by incubation with 10 μg/mL of DIZE. After 6 h, 5 μg/mL LPS was added to the cells and cultured for 24 h. The supernatants were collected and stored at −20 °C.

To investigate the involvement of MAPK and NF-κB pathways in LPS-induced cytokine expression, ARPE-19 cells were seeded into 24-well plates to become confluent. After being serum-free starved for 24 h, cells were pre-incubated for 30 min in serum-free medium with p38 inhibitor SB203580, extracellular signal-regulated kinase 1/2 (ERK1/2) inhibitor PD98059, and JNK inhibitor SP600125 (all from Cell Signaling Technology, Beverly, MA) and NF-κB inhibitor BAY 11-7082 (Sigma-Aldrich) at the concentration of 10 μM. Subsequently, 5 μg/mL LPS was added to the medium. After 24 h of incubation, the supernatants were collected and stored at −20 °C.

### ACE2 small-interfering RNA (siRNA) transfection and LPS treatment

A siRNA-targeting specific sequence of ACE2 and a negative control scrambled siRNA, which was not homologous to any gene, were synthesized by GenePharma (Shanghai, China). The transfection was performed as previously described [35]. The sequences of these siRNAs were shown in Table 1. The ARPE-19 cells and primary human RPE were seeded in 12-well plates (1–2 × 10^5^ cells/well) and cultured in 1 mL DMEM culture medium containing 10 % FBS without antibiotics until the cells were 50~60 % confluent. Before transfection, cells were serum-free starved for 12 h. Lipofectamine® 2000 Transfection Reagent (Invitrogen, Carlsbad, CA) was used to perform the transfection. The siRNA complex was pre-mixed according to the manufacturer’s instructions and added to the 12-well plates. The final concentration of ACE2 and scrambled siRNA was 100 nM per well as the previous study [36]. Six hours after transfection, the medium was changed to fresh serum-free culture medium without antibiotics. The cells were incubated with DIZE (10 μg/mL) for 6 h, and then stimulated with LPS (5 μg/mL) for 24 h. The cell samples were collected for subsequent assays.

**Table 1:** Sequences of small-interfering RNA

Gene	Forward	Reverse
ACE2-siRNA	5′-CCA UCU ACA GUA CUG GAA A dTdT-3′	5′-UUU CCA GUA CUG UAG AUG G dTdT-3′
Scrambled siRNA	5′-UUC UCC GAA CGU GUC ACG U dtdt-3′	5′-ACG UGA CAC GUU CGG AGA A dtdt-3′

### Total RNA extraction and real-time PCR

Total RNA was extracted from hRPE and ARPE-19 cells by using Trizol Reagent (Invitrogen) according to the manufacturer’s instructions. Complementary DNA (cDNA) was generated using PrimeScript RT reagent Kit (Takara Biotechnology, Dalian, China). Real-time PCR (SYBR Green) was performed according to manufacturer’s instruction with a sequence detection system (ABI Prism 7500; Applied Biosystems, Foster City, CA). The real-time PCR amplification was performed in a volume of 20 μL using all-in-one quantitative PCR mix (Takara Biotechnology). The cycling protocol consisted of 1 cycle of 10 min at 95 °C followed by 40 cycles of 95 °C for 15 s and 60 °C for 1 min. The mRNA expression was normalized to the endogenous reference gene β-actin. Relative quantification was achieved by the comparative 2^−∆∆ct^ method as described [14]. The sequences of the primers are shown in Table 2.

**Table 2:** Sequences of the primers for real-time PCR

Gene	Accession number	Forward	Reverse
IL-6	NM_000600.3	5′-AGTGAGGAACAAGCCAGAGC-3′	5′-CAGGGGTGGTTATTGCATCT-3′
IL-8	NM_000584.3	5′-GACATACTCCAAACCTTTCCACCC-3′	5′-CCAGACAGAGCTCTCTTCCATCAG-3′
MCP-1	NM_002982.3	5′-CTCATAGCAGCCACCTTCATTC-3′	5′-TCACAGCTTCTTTGGGACACTT-3′
ACE2	NM_021804.2	5′-CATTGGAGCAAGTGTTGGATCTT-3′	5′-GAGCTAATGCATGCCATTCTC A-3′
AT1R	NM_032049.3	5′-GATGATTGTCCCAAAGCTGG-3′	5′-TAGGTAATTGCCAAAGGGCC-3′
AT2R	NM_000686.4	5′-CCTCGCTGTGGCTGATTTACTC-3′	5′-CTTTGCACATCACAGGTCCAA-3′
β-actin	NM_001101.3	5′-GGATGCAGAAGGAGATCACTG-3′	5′-CGATCCACACGGAGTACTTG-3′

### Enzyme-linked immunosorbent assay

The concentrations of inflammatory cytokines IL-6, IL-8, and MCP-1 (R&D Systems, Minneapolis, Minnesota, CA), Ang II (Abcam, Cambridge, MA) and Ang-(1-7) (Cloud-Clone, Houston, MA) were determined using the ELISA kits for human according to the manufacturer’s instructions. The absorbance at 450 nm wavelength was measured using a multifunction microplate reader (Molecular Devices).

### Western blot analysis

Cells were washed with cold PBS for three times and scraped off with RIPA lysis buffer (Beyotime) containing 50 mM Tris-HCl (pH 7.4), 150 mM NaCl, 1 % Triton X-100, 1 % sodium deoxycholate, 0.1 % SDS, 2 mM EDTA, and 100 M phenylmethylsulfonyl fluoride. The cellular lysate was centrifuged and supernatant was collected. Protein concentration was detected by bicinchoninic acid assay kit (Beyotime). Samples were denatured with Laemmli loading buffer at 100 °C for 5 min. Eighty microgram samples were loaded to an 8 to 12 % SDS-polyacrylamide gel. Pre-stained markers (Beyotime) were used to estimate molecular weight. Then proteins were transferred to polyvinylidene difluoride membranes (Millipore, Bedford, MA), blocked by 5 % non-fat skim milk or 5 % bovine serum albumin-Tris-buffered saline supplemented with Tween 20, the membranes were incubated with primary antibodies against ACE2 (1:500, Abcam, Cambridge, MA), p-p38, p38, ERK1/2 (1:1000; Cell Signaling), p-JNK, JNK (1:500; Cell Signaling), p-ERK1/2 (1:2000; Cell Signaling), p-IκB-α (1:50, Santa Cruz) and β-actin (1:100; Abcam) overnight at 4 °C. Membranes were washed and incubated with horseradish peroxidase-conjugated secondary antibody (1:2000 or 1:3000; Abcam) at 37 °C for 1 h. Bands were visualized by ECL kit (Advansta, Menlo Park, CA), and band densitometry was performed using ImageJ software (Bethesda, MD). The band intensity of p-IκB-α was normalized to β-actin, and the band intensity of p-p38, p-JNK, and p-ERK1/2 was normalized to p38, JNK, and ERK1/2, respectively.

### Statistical analysis

Results were expressed as mean ± SEM. Experimental data of multiple groups were analyzed by one-way ANOVA followed by Bonferroni correction. *p* < 0.05 was considered to be significantly different for all experiments.

## Results

### Determine the optimum concentration and incubation time of DIZE in ARPE-19 cells

**Fig. 1:**
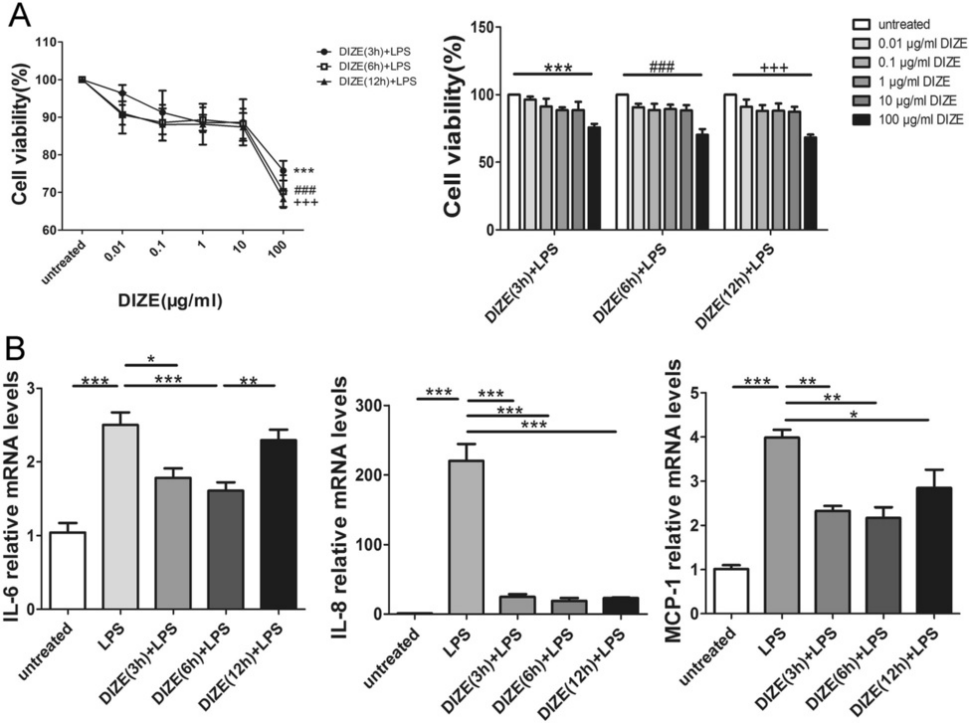
The optimum concentration and incubation time of DIZE in cultured ARPE-19 cell line. **a** The cell viability was measured by CCK-8 assay. ARPE-19 cells were treated with 0.01, 0.1, 1, 10, and 100 μg/mL of DIZE for 3, 6, and 12 h and then stimulated with LPS for 24 h. (***p < 0.001, ^###^ *p* < 0.001, ^+++^ *p* < 0.001, *n* = 6, “*asterisk*” “*number sign*” “*plus sign*” indicated pre-incubated with 100 μg/mL of DIZE for 3, 6, and 12 h versus time-matched untreated cells, respectively). **b** ARPE-19 cells were pre-treated with 10 μg/mL of DIZE for 3, 6, and 12 h, following stimulated with LPS for 24 h. The mRNA expression of IL-6, IL-8, and MCP-1 in the DIZE-treated groups were compared with the LPS group by real-time PCR. All data are expressed as mean ± SEM. (***p < 0.001, **p < 0.01, *p < 0.05, n = 4 ~ 6)

The effect of DIZE on the viability of ARPE-19 cells was tested with different concentrations of DIZE. Pre-incubation of DIZE with the concentrations of 0.01, 0.1, 1, and 10 μg/mL for 3, 6, and 12 h followed by stimulation with LPS for 24 h had no noticeable influence on the viability of ARPE-19 cells compared to the untreated controls. However, pre-incubation with 100 μg/mL of DIZE for 3, 6, and 12 h followed by stimulation with LPS for 24 h significantly decreased the cell viability (Fig. 1 a). Therefore, a concentration of 10 μg/mL of DIZE was used in subsequently experiments. ARPE-19 cells were pre-treated with 10 μg/mL DIZE for 3, 6, 12 h, then stimulated with 5 μg/mL of LPS for 24 h. Real-time PCR was performed to measure the levels of inflammatory cytokines. The mRNA levels of IL-6, IL-8, and MCP-1 were significantly decreased in the cells pre-incubated with DIZE for 3, 6, and 12 h at concentration 10 μg/mL. Based on the changes of cytokines’ mRNA, we chose 6 h as the pre-incubation time for DIZE in following experiment (Fig. 1b ,***p < 0.001, **p < 0.01, *p < 0.05, *n* = 4 ~ 6).

### Identification of human retinal pigment epithelium (hRPE) and ARPE-19 cell line

**Fig. 2:**
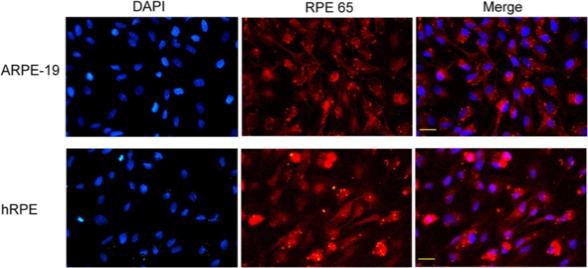
Identification of cutured hRPE and ARPE-19 cells. The RPE65 is a RPE-specific protein. It was used to identify whether the primary cells isolated from the donor eyes, and the cell line were both derived from RPE. Merged images showed that the RPE65 was localized in the cytoplasm of the cells with DAPI-positive nucleus in both the primary cultured hRPE and the ARPE-19. The RPE65-positive cells were counted in five non-overlapping fields for each type of cell under a fluorescence microscope. The ratio of RPE65-positive cell was calculated as the percentage of RPE65-positive cells in the DAPI-positive cells. The ratio was 96.1 % ± 1.4 % in ARPE-19 cells, and 91.3 % ± 4.7 % in the hRPE cells. The scale bar 100 μm, magnification ×200

The antibody of the RPE-specific protein RPE65 was used to identify the hRPE and the ARPE-19 cells. In the primary human PRE cells, RPE65 was localized in the cytoplasm around the DAPI-positive nucleus (Fig. 2). The ARPE-19 cells presented same pattern. It indicated that both the primary and the ARPE-19 cells were RPE-derived cells. The RPE65-positive cells were counted in five non-overlapping fields for each type of cell under a fluorescence microscope. The percentage of the RPE65-positive cell in DAPI-positive cells was calculated. The percentage was 96.1 % ± 1.4 % in ARPE-19 cells, and 91.3 % ± 4.7 % in hRPE cells.

### DIZE increased, whereas ACE2-siRNA decreased ACE2 expression in hRPE and ARPE-19 cells

**Fig. 3:**
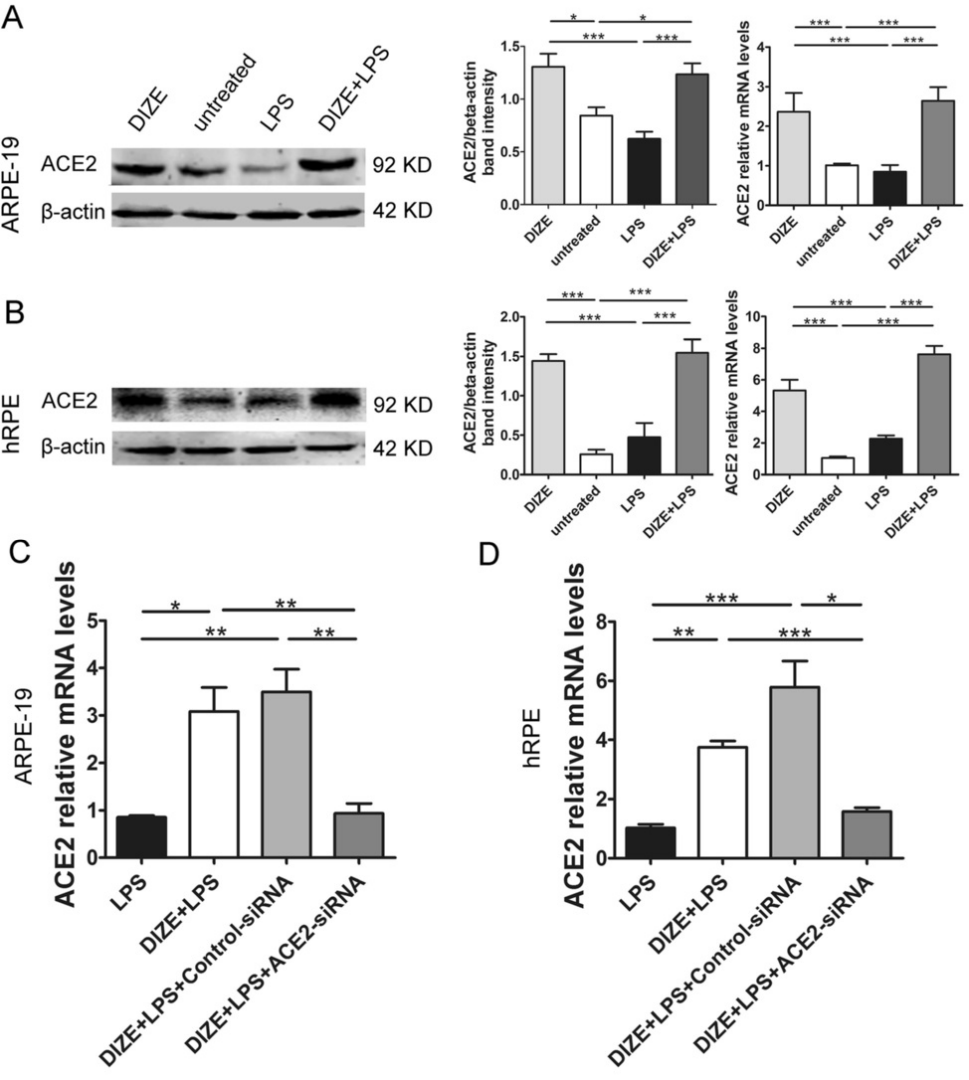
Expression of ACE2 in DIZE pre-treated hRPE and ARPE-19 cells followed by LPS stimulation. The mRNA and protein expressions of ACE2 detected by real-time PCR and Western blotting showed that the ACE2 levels increased in the DIZE group and the DIZE+LPS group when compared with the untreated group and the LPS-treated group in both ARPE-19 cells (**a**) and hRPE (**b**). There was no significant difference between the untreated group and the LPS group. The band intensity of ACE2 was normalized to β-actin. The mRNA level of ACE2 was detected by real-time PCR in both ARPE-19 cells (**c**) and hRPE (**d**), which were transfected with ACE2-siRNA or the negative control scrambled siRNA. All data are expressed as mean ± SEM. (***p < 0.001, **p < 0.01, *p < 0.05, *n* = 4)

To investigate the effect of DIZE on the expression of ACE2 in hRPE and ARPE-19 cells, real-time PCR and Western blotting were performed. The mRNA expression of ACE2 significantly increased in the DIZE+LPS and DIZE group compared with the untreated group. There was no statistical difference between the untreated group and the LPS group in both ARPE-19 cells (Fig. 3a) and hRPE cells (Fig. 3b). The protein level of ACE2 was consistent with the mRNA expression. The protein expression of ACE2 increased remarkably in the DIZE group and in the DIZE+LPS group. There was no significantly difference between the LPS group and the untreated group in both ARPE-19 cells (Fig. 3a) and hRPE cells (Fig. 3b). In ARPE-19 cells (Fig. 3c) and in hRPE cells (Fig. 3d), the mRNA expression of ACE2 was remarkably decreased by ACE2-siRNA when compared with the DIZE+LPS group and the DIZE+LPS group treated with the negative control scrambled siRNA. (***p < 0.001, **p < 0.01, *p < 0.05, *n* = 4).

### DIZE downregulated the overproduction of pro-inflammatory cytokines in LPS-stimulated hRPE and ARPE-19 cells

**Fig. 4:**
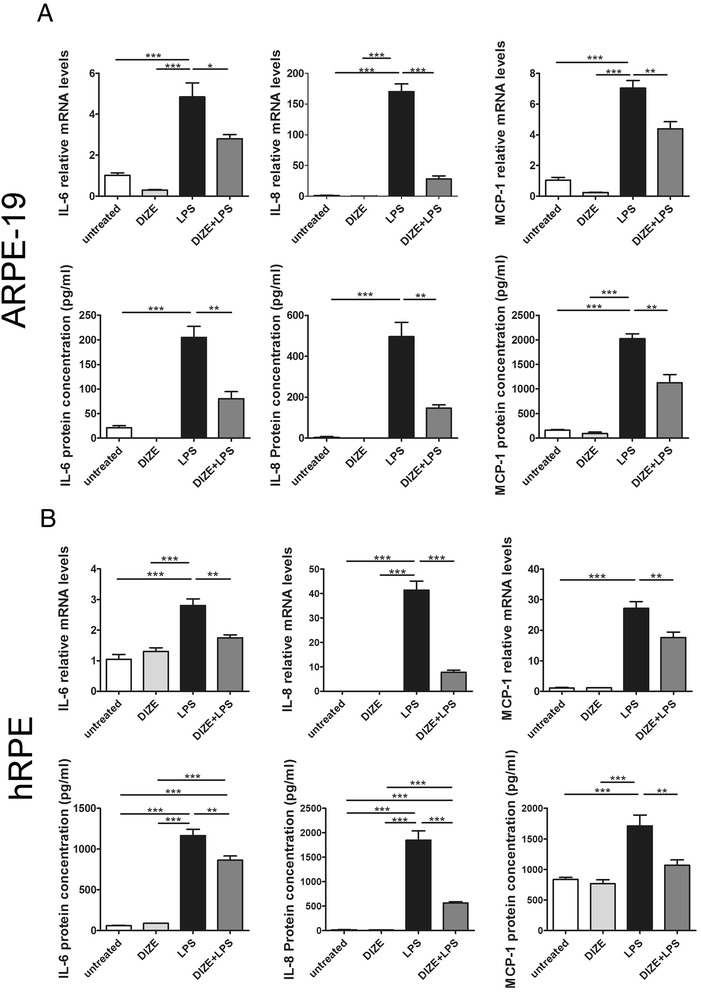
The mRNA and protein expressions of pro-inflammatory cytokines in DIZE pre-treated hRPE and ARPE-19 cells followed by LPS stimulation. Real-time PCR and ELISA were conducted to determine the production of IL-6, IL-8, and MCP-1 in ARPE-19 cells (**a**) as well as hRPE (**b**). Exposure to LPS for 24 h caused a significant overproduction of IL-6, IL-8, and MCP-1 in the ARPE-19 cells and hRPE. Whereas overproduction of the cytokines was markedly inhibited by DIZE. IL-6 and IL-8 protein levels were undetectable in the DIZE group. All data are expressed as mean ± SEM. (***p < 0.001, **p < 0.01, *p < 0.05, *n* = 4)

To investigate the effect of DIZE on the overproduction of pro-inflammatory cytokines in hRPE and ARPE-19 cells stimulated with LPS, cells were pre-treated with or without 10 μg/mL of DIZE for 6 h and then stimulated with LPS for 24 h. The mRNA and protein expression of the inflammatory cytokines was determined by real-time PCR and ELISA assay. The levels of IL-6, IL-8, and MCP-1 in the cells exposed to LPS were significantly higher than the control group. Overexpression of IL-6, IL-8, and MCP-1 induced by LPS was significantly inhibited by DIZE. DIZE resulted in a reduction of mRNA expression of IL-6, IL-8, and MCP-1 by 1.66-, 7.6-, and 1.71-fold in the DIZE+LPS group compared with the LPS group in the ARPE-19 cells (Fig. 4a) and a reduction by 1.35-, 3.28-, and 1.6-fold in the hRPE (Fig. 4b). In the DIZE+LPS group, the protein levels of IL-6, IL-8, and MCP-1 significantly decreased (2.55-, 3.38-, 1.79-fold) than the LPS group in the ARPE-19 cells (Fig. 4a) and 1.6-, 5.31-, and 1.54-fold decreased in the hRPE (Fig. 4b). There was no difference of IL-6, IL-8, and MCP-1 expression between the untreated group and the DIZE group at both mRNA and protein levels. (***p < 0.001, **p < 0.01, *p < 0.05, *n* = 4).

### A small interfering RNA of ACE2 and an Ang-(1-7) antagonist A779 abrogated the anti-inflammatory effect of DIZE

**Fig. 5:**
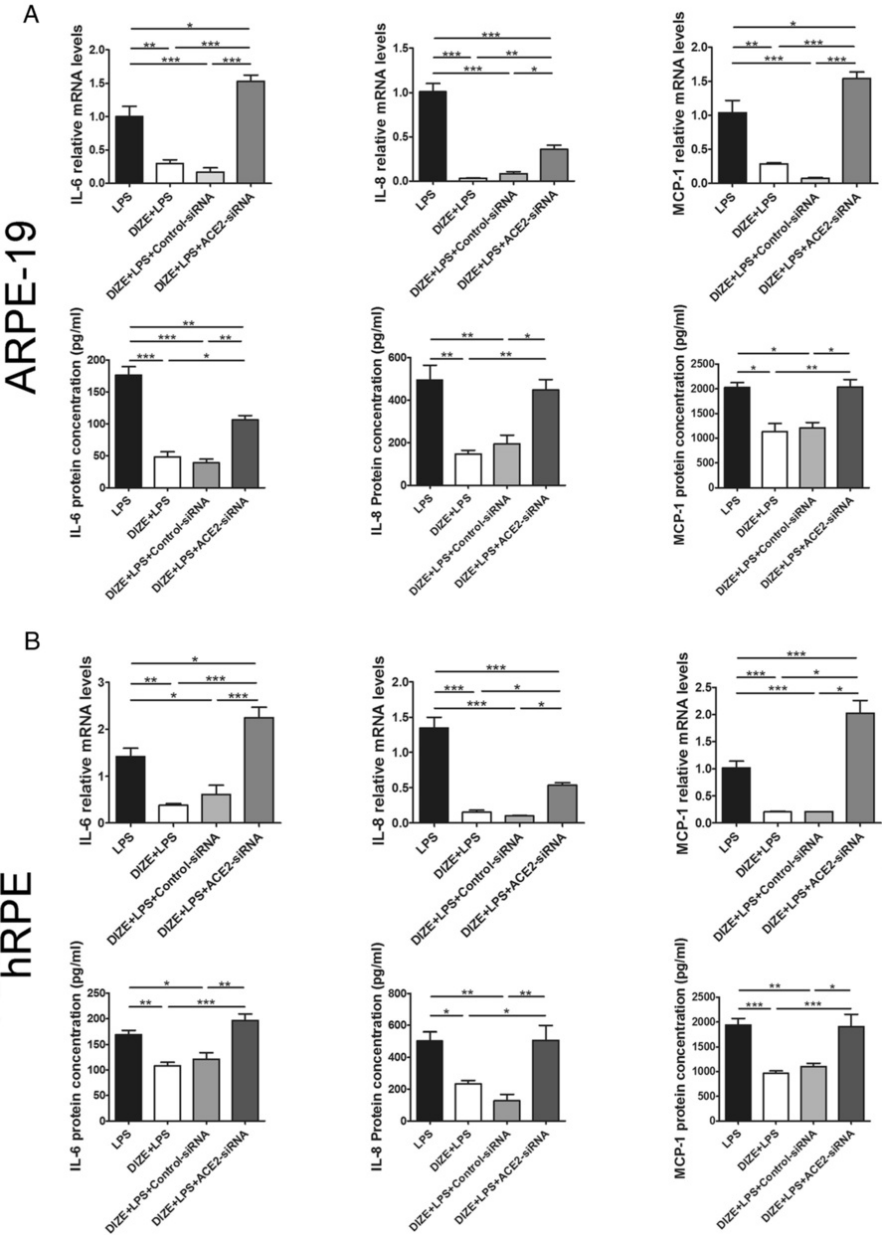
Effect of ACE2-siRNA on the production of inflammatory cytokines in ARPE-19 cells and hRPE. IL-6, IL-8, and MCP-1 were detected by real-time PCR and ELISA assay in both ARPE-19 cells (**a**) and hRPE (**b**). DIZE reduced the expression of IL-6, IL-8, and MCP-1 in LPS-treated cells at both mRNA and protein levels, while ACE2-siRNA reversed this effect. All data are expressed as mean ± SEM (***p < 0.001, **p < 0.01, *p < 0.05, *n* = 4)

**Fig. 6:**
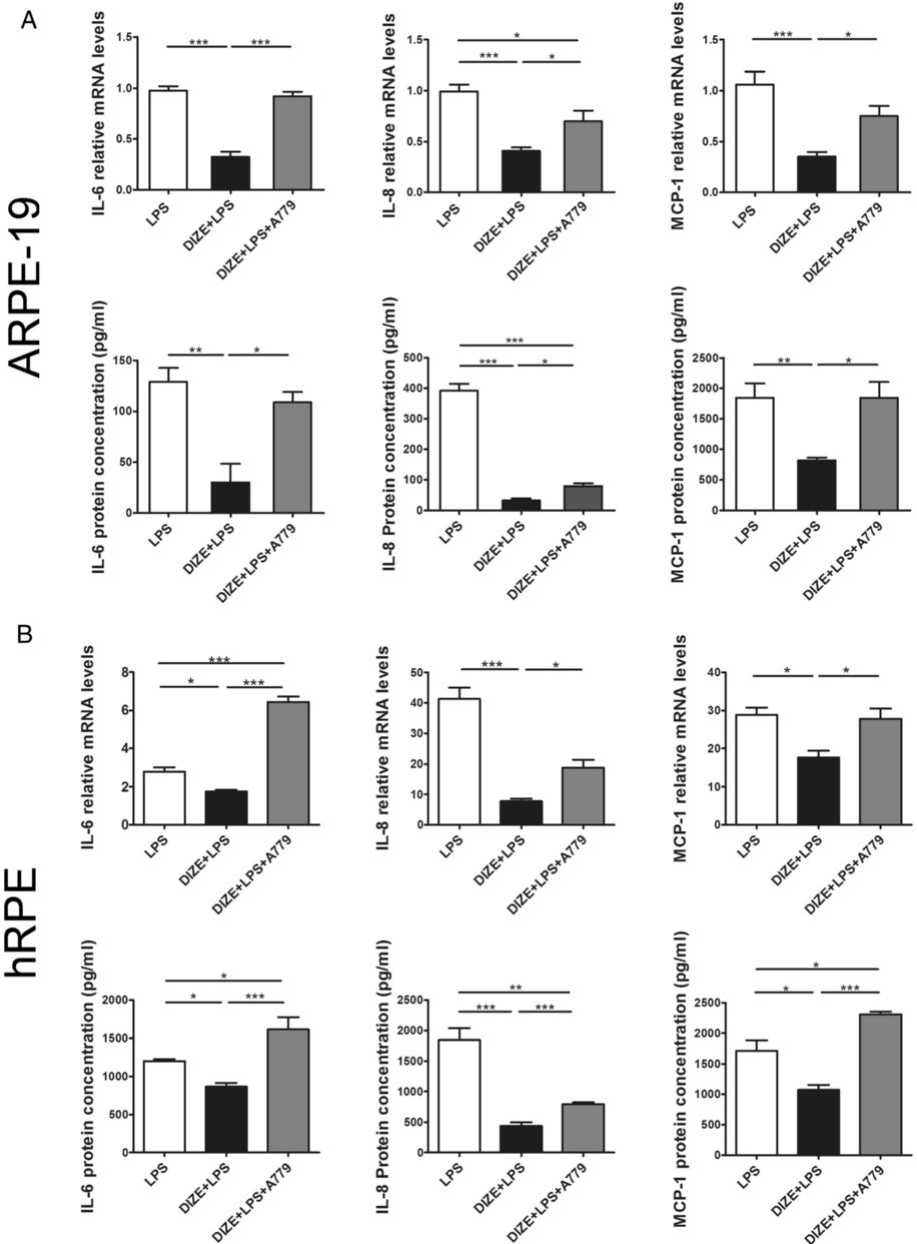
Effect of A779 on the production of inflammatory cytokines in ARPE-19 cells and hRPE. IL-6, IL-8, and MCP-1 were detected by real-time PCR and ELISA assay in both ARPE-19 cells (**a**) and hRPE (**b**). In LPS-treated cells, DIZE reduced the expression of IL-6, IL-8, and MCP-1, while A779 reversed this effect. All data are expressed as mean ± SEM (***p < 0.001, **p < 0.01, *p < 0.05, *n* = 4)

To investigate whether DIZE exerted the protective role by activating ACE2 and the involvement of the ACE2/Ang-(1-7)/Mas axis, we transfected the ARPE-19 cells and hRPE with ACE2-siRNA or treated them with A779. Both ACE2-siRNA and A779 offset the anti-inflammatory effect of DIZE. They reversed the reduction of IL-6, IL-8, and MCP-1 at both mRNA and protein levels in the ARPE-19 cells (Figs. 5 and 6a) and the hRPE (Figs. 5, 6b) (***p < 0.001, **p < 0.01, *p < 0.05, *n* = 4).

### DIZE decreased the expression of Ang II, AT1R but increased Ang-(1-7), while ACE2-siRNA and A779 reversed these effects

**Fig. 7:**
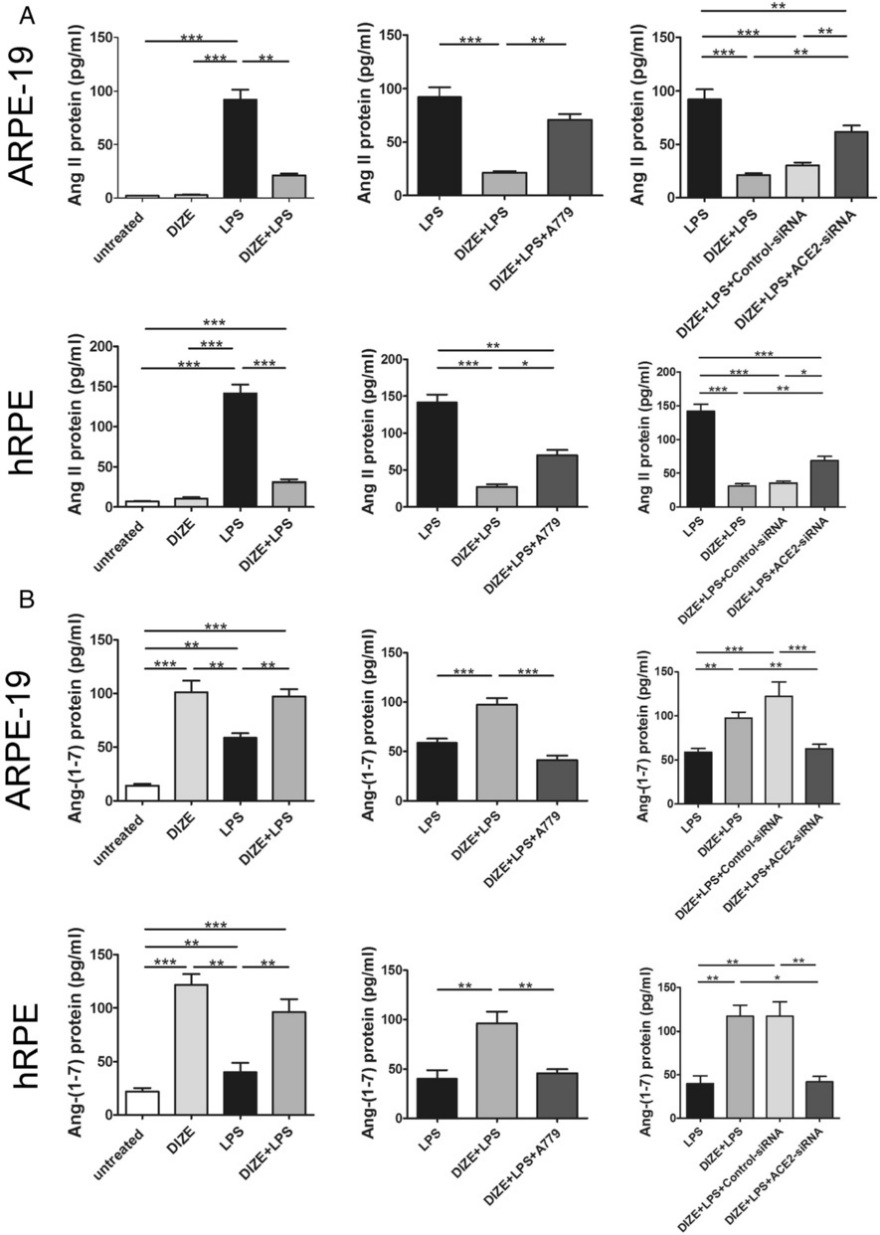
Effect of DIZE, A779, and ACE2-siRNA on the expression of Ang II and Ang-(1-7) in ARPE-19 cells and hRPE. The protein expression of Ang II and Ang-(1-7) was detected by ELISA assay in both ARPE-19 cells and hRPE. DIZE reduced the expression of Ang II in response to LPS, while A779 and ACE2-siRNA reversed this effect (**a**). DIZE increased the expression of Ang-(1-7) compared with LPS-treated cells, while A779 and ACE2-siRNA reversed this effect (**b**). All data are expressed as mean ± SEM (***p < 0.001, **p < 0.01, *p < 0.05, *n* = 4 ~ 6)

**Fig. 8:**
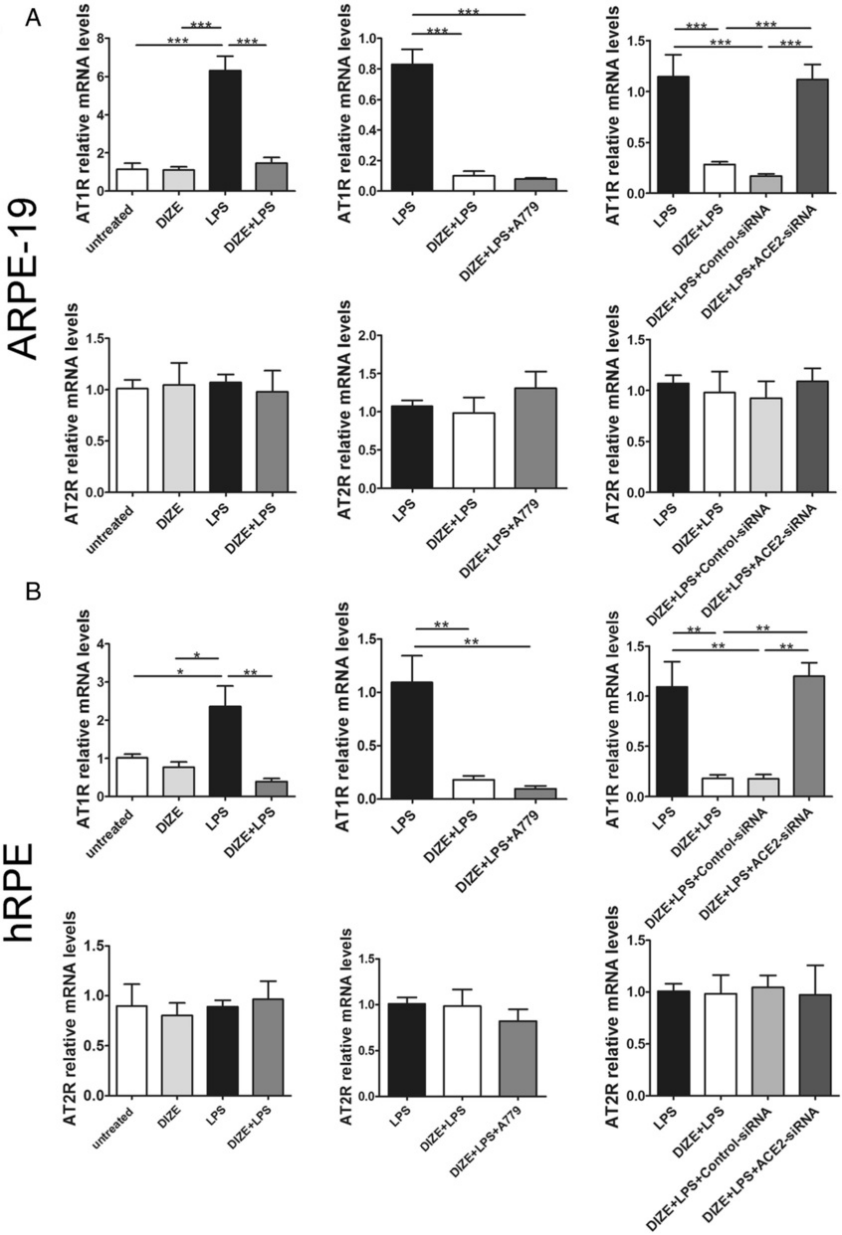
Effect of DIZE, A779, and ACE2-siRNA on the expression of AT1R and AT2R in ARPE-19 cells and hRPE. The mRNA level of AT1R and AT2R was detected by real-time PCR in both ARPE-19 cells and hRPE. DIZE reduced the expression of AT1R in response to LPS, while ACE2-siRNA reversed this effect. A779 did not affect the expression of AT1R. There was no significant difference of AT2R expression among each group in ARPE-19 cells (**a**) and hRPE (**b**). All data are expressed as mean ± SEM (***p < 0.001, **p < 0.01, *p < 0.05, *n* = 4)

Ang II is the main effector of RAS. Increase of Ang II could exert a series of detrimental effects mediated by its receptor AT1R. However, ACE2 could cleave Ang II to generate Ang-(1-7), which is a member of the protective axis of RAS: ACE2/Ang-(1-7)/Mas. To investigate whether the protective role of DIZE is mediated by activating ACE2 and by switching Ang II to Ang-(1-7), we detected the expression of Ang II and Ang-(1-7) by ELISA. DIZE significantly reduced the Ang II level compared with the LPS-treated ARPE-19 cells and hRPE. While ACE2-siRNA and A779 reversed the inhibition effect of DIZE (Fig. 7a). On the contrary, DIZE resulted in dramatically increase of Ang-(1-7) compared with the LPS treated ARPE-19 cells and hRPE. Whereas ACE2-siRNA and A779 reversed the effect of DIZE (Fig. 7b). The protein fold changes of Ang II and Ang-(1-7) were shown in Table 3. Moreover, studies have shown that AT1R increases in response to the inflammation stimuli. AT2R might display a counteraction role of AT1R. Thus, we investigated the effect of ACE2 on the expression of AT1R and AT2R. LPS treatment significantly increased the expression of AT1R, while DIZE reduced it. ACE2-siRNA abolished the inhibition effect of DIZE. It increased AT1R compared with DIZE+LPS group in both ARPE-19 cells (Fig. 8a) and hRPE (Fig. 8b). However, A779 did not abrogate the effect of DIZE on the expression of AT1R. Furthermore, there was no significance of AT2R expression in each group (Fig. 8a, b).

**Table 3:** The protein fold changes (vs. the LPS group)

Protein	Cell line	Group	Change	Fold change
Ang II	ARPE-19	DIZE	↓	32.89
		DIZE+LPS	↓	4.33
		DIZE+LPS+A779	↓	1.30
		DIZE+LPS+control-siRNA	↓	3.04
		DIZE+LPS+ACE2siRNA	↓	1.50
	hRPE	DIZE	↓	13.56
		DIZE+LPS	↓	4.57
		DIZE+LPS+A779	↓	2.02
		DIZE+LPS+control-siRNA	↓	4.00
		DIZE+LPS+ACE2siRNA	↓	2.06
Ang (1-7)	ARPE-19	DIZE	↑	1.73
		DIZE+LPS	↑	1.66
		DIZE+LPS+A779	↓	1.42
		DIZE+LPS+control-siRNA	↑	2.09
		DIZE+LPS+ACE2siRNA	↑	1.07
	hRPE	DIZE	↑	2.62
		DIZE+LPS	↑	2.30
		DIZE+LPS+A779	↑	1.10
		DIZE+LPS+control-siRNA	↑	2.88
		DIZE+LPS+ACE2siRNA	↑	1.10

### Inhibitors of the MAPK and NF-κB pathways reduced the overproduction of inflammatory cytokines in ARPE-19 cells stimulated with LPS

**Fig. 9:**
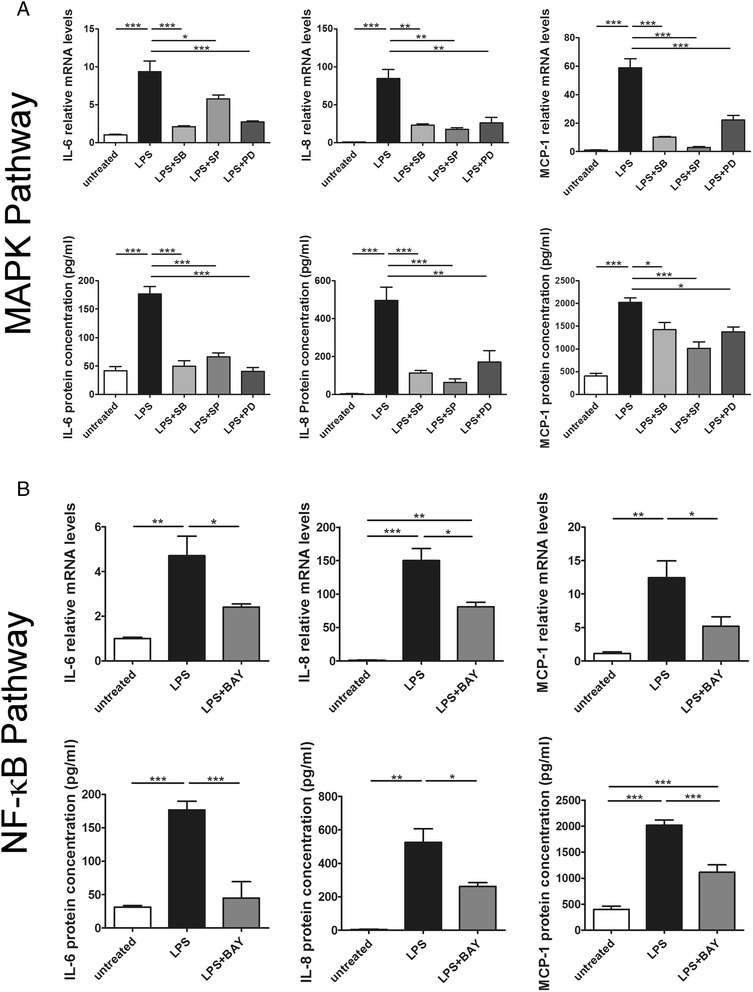
Effect of the MAPK and NF-κB inhibitors on the mRNA and protein expression of inflammatory cytokines in LPS-stimulated ARPE-19 cells. The expression of IL-6, IL-8, and MCP-1 was detected by real-time PCR and ELISA assay in the MAPK pathway inhibitor-treated cells (**a**) and NF-κB pathway inhibitor-treated cells (**b**). The p38 inhibitor (SB203580, SB), JNK inhibitor (SP600125, SP), and ERK1/2 inhibitor (PD98059, PD) significantly decreased the production of IL-6, IL-8, and MCP-1 in the ARPE-19 cells stimulated with LPS at both mRNA and protein levels (**a**). The NF-κB inhibitor (BAY 11-7082, BAY) remarkably reduced the production of IL-6, IL-8, and MCP-1 in LPS-treated group at both mRNA and protein levels (**b**). All data are expressed as mean ± SEM (***p < 0.001, **p < 0.01, *p < 0.05, *n* = 4)

To investigate whether the signaling pathways were involved in LPS-induced cytokine overproduction in ARPE-19 cells, the selective inhibitors of p38 (SB203580, SB), ERK1/2 (PD98059, PD), JNK (SP600125, SP), and NF-κB (BAY 11-7082, BAY) were applied 30 min before LPS administration. SB, PD, SP, and BAY significantly inhibited the mRNA and protein overproduction of IL-6, IL-8, and MCP-1. Compared with the LPS group, the mRNA expression of IL-6, IL-8, and MCP-1 in the LPS+SB group decreased remarkably by 4.41-, 3.66-, and 5.74-fold, respectively (Fig. 9a). The protein level of IL-6, IL-8, and MCP-1 in the LPS+SB group reduced by 3.56-, 4.39-, and 1.42-fold when compared with the LPS group (Fig. 9a). The mRNA expression of IL-6, IL-8, and MCP-1 in the LPS+SP group decreased by 1.63-, 4.80-, and 11.69-fold, respectively, when compared with the LPS group (Fig. 9a). The protein level of IL-6, IL-8, and MCP-1 in the LPS+SP group was reduced by 2.67-, 7.83-, and 2.00-fold compared with the LPS group (Fig. 9a). PD treatment led to significant decrease of the inflammatory cytokines’ mRNA. The mRNA expression of IL-6, IL-8, and MCP-1 decreased by 3.43-, 3.26-, and 2.66-fold, respectively (Fig. 9a), and there was 4.36-, 2.90-, and 1.47-fold reduction of IL-6, IL-8, and MCP-1 protein in the LPS+PD group compared with the LPS group (Fig. 9a). The mRNA expression of IL-6, IL-8, and MCP-1 in the LPS+BAY group decreased by 1.95-, 1.85-, and 2.39-fold, respectively, compared with the LPS group (Fig. 9b). The protein level of IL-6, IL-8, and MCP-1 in the LPS+BAY group was reduced by 2.60-, 2.01-, and 1.73-fold compared with the LPS group (Fig. 9b) (***p < 0.001, **p < 0.01, *p < 0.05, *n* = 4 ~ 6). The mRNA and protein fold changes of pro-inflammatory cytokines were shown in Table 4.

**Table 4:** The mRNA and protein fold changes of pro-inflammatory cytokines (vs. the LPS group)

Group	Cytokines	mRNA fold change	Protein fold change
LPS+SB	IL-6	4.41	3.56
	IL-8	3.66	4.39
	MCP-1	5.74	1.42
LPS+SP	IL-6	1.63	2.67
	IL-8	4.80	7.83
	MCP-1	11.69	2.00
LPS+PD	IL-6	3.43	4.36
	IL-8	3.26	2.90
	MCP-1	2.66	1.47
LPS+BAY	IL-6	1.95	2.60
	IL-8	1.85	2.01
	MCP-1	2.39	1.73

### DIZE downregulated the phosphorylation of MAPKs and NF-κB in ARPE-19 cells stimulated with LPS

**Fig. 10:**
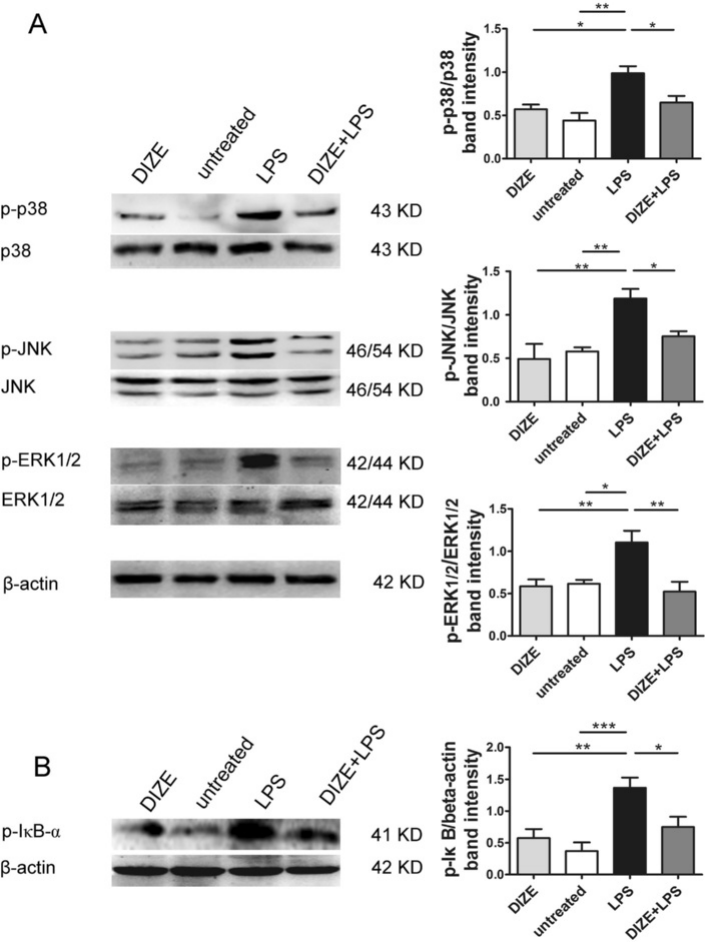
Effect of DIZE on the phosphorylation levels of MAPKs and the activation of NF-κB in ARPE-19 cells stimulated with LPS. **a** Western blotting was used to determine the protein levels of p38 MAPK, JNK, ERK1/2 and their phosphorylated forms (p-p38, p-JNK, and p-ERK1/2). **b** Activation of NF-κB pathway was indicated by p-IκB-α, an indicator for the activation of NF-κB. The expressions of p-p38, p-JNK, and p-ERK1/2 were normalized to p38, JNK, and ERK1/2, respectively. The band intensity of p-IκB-α was normalized to β-actin. DIZE treatment resulted in remarkably reduction of phosphorylation of p38, JNK, ERK1/2, and IκB-α compared with the LPS-stimulated group. All data are expressed as mean ± SEM (***p < 0.001, **p < 0.01, *p < 0.05, *n* = 3 ~ 4)

**Fig. 11:**
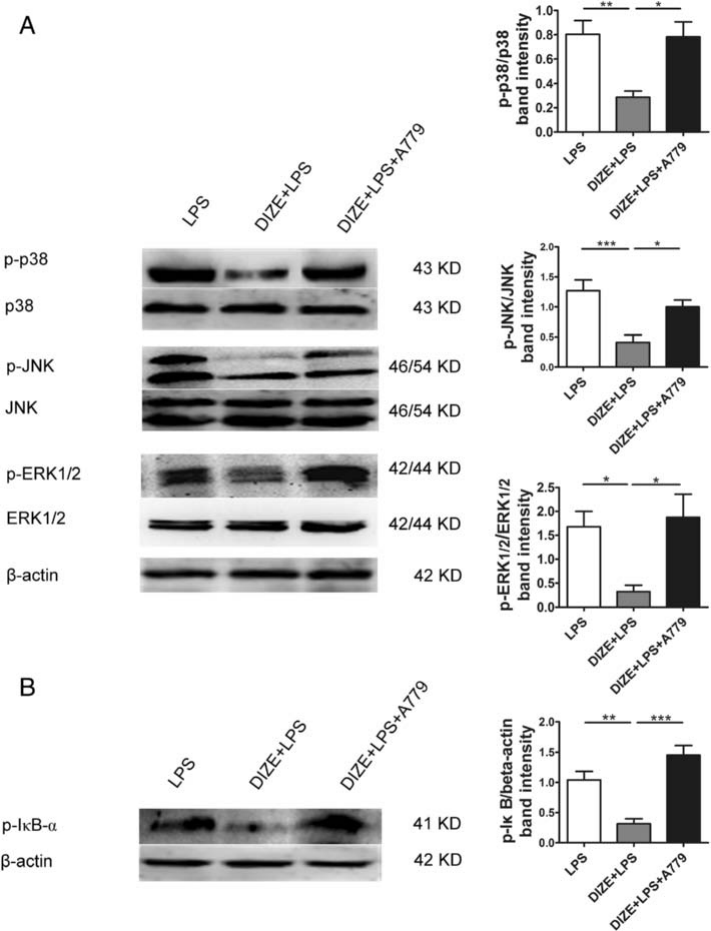
Effect of A779 on the phosphorylation of MAPKs and the activation of NF-κB in ARPE-19. Western blotting was applied to determine the protein levels of p-p38, p-JNK, p-ERK1/2, p38, JNK, ERK1/2 (**a**) and p-IκB-α, an indicator for the activation of NF-κB (**b**). The band intensity of p-p38, p-JNK, and p-ERK1/2 was normalized to p38, JNK, and ERK1/2, respectively. The band intensity of p-IκB-α was normalized to β-actin. DIZE reduced the phosphorylation of p38, JNK, and ERK1/2 and IκB-α induced by LPS, while A779 abrogated DIZE’s effect. All data are expressed as mean ± SEM (***p < 0.001, **p < 0.01, *p < 0.05, *n* = 3 ~ 4)

**Fig. 12:**
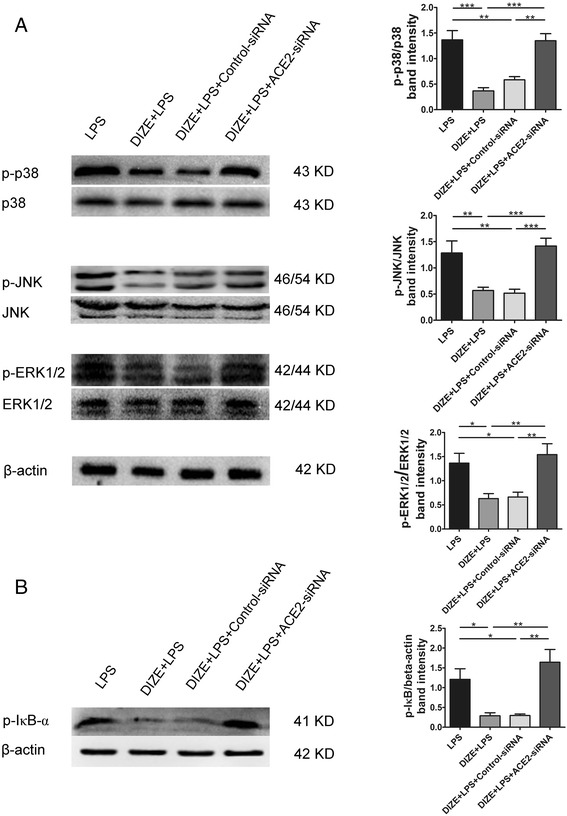
Effect of ACE2-siRNA on the phosphorylation of MAPKs and the activation of NF-κB in ARPE-19. The protein levels of p-p38, p-JNK, p-ERK1/2, p38, JNK, ERK1/2 (**a**) and p-IκB-α (**b**) were determined by Western blotting. The band intensity of p-p38, p-JNK, and p-ERK1/2 was normalized to p38, JNK, and ERK1/2, respectively. The band intensity of p-IκB-α was normalized to β-actin. DIZE reduced the phosphorylation of p38, JNK, and ERK1/2 and IκB-α induced by LPS, while ACE2-siRNA abolished DIZE’s effect. All data are expressed as mean ± SEM (***p < 0.001, **p < 0.01, *p < 0.05, *n* = 3 ~ 4)

MAPKs and NF-κB signaling pathways are critical in regulating the production of pro-inflammatory cytokines in various cells [37–39]. To further explore the underlying mechanisms by which DIZE suppressed LPS-induced pro-inflammatory cytokine production, we tested the protein expression of phosphorylated transcription inhibition factor-κB-α (p-IκB-α), an indicator of the activation of NF-κB, phosphorylated p38 (p-p38), p-JNK, and p-ERK1/2. Western blotting results showed that the expression of p-p38, p-JNK, p-ERK1/2, and p-IκB-α were significantly higher in the LPS-stimulated group compared with the DIZE group and the untreated group. DIZE treatment (DIZE+LPS group) resulted in remarkably reduction in protein expression of p-p38, p-JNK, p-ERK1/2, and p-IκB-α compared with the LPS-stimulated group (Fig. 10a, b). DIZE exerted anti-inflammatory effect by inhibiting the activation of MAPK and NF-κB pathways in cells stimulated with LPS. (***p < 0.001, **p < 0.01, *p < 0.05, *n* = 3 ~ 4). A779 abrogated the protective role of DIZE via offsetting the inhibition of the MAPK and the NF-κB pathways at protein level (Fig. 11). ACE2-siRNA also abolished the beneficial effect of DIZE by inhibiting the MAPK and the NF-κB pathways at the protein level (Fig. 12) (***p < 0.001, **p < 0.01, *p < 0.05, *n* = 3 ~ 4).

## Discussion

We investigated the effect of DIZE on LPS-induced inflammatory response in hRPE and ARPE-19 cells. We showed that DIZE reduced the overexpression of IL-6, IL-8, and MCP-1 in response to LPS stimulation, and the effect is associated with the activation of the ACE2/Ang-(1-7)/Mas axis. DIZE treatment also decreased the expression of Ang II, AT1R but increased Ang-(1-7). Meanwhile, we found that the MAPK and NF-κB pathways were involved in the LPS-induced overproduction of IL-6, IL-8, and MCP-1. Whereas, pre-treatment with DIZE downregulated phosphorylation of p38MAPK, JNK, ERK1/2 and inhibited NF-κB pathway. On the other hand, the ACE2-siRNA and an Ang-(1-7) antagonist reversed the protective effect of DIZE. Taking together, we suggest that the protective effects of DIZE on LPS-induced inflammatory response were mediated by activating the ACE2/Ang-(1-7)/Mas axis and by inhibiting the MAPK and NF-κB pathways.

RAS is considered traditionally a blood-pressure-regulating system. Whereas, increasing evidence suggests that it is also important in regulating inflammatory responses. Hyperactivation of the ocular intrinsic RAS contributes to an upregulation of ACE/Ang II/AT1R axis and a downregulation of ACE2/Ang-(1-7)/Mas axis. It has been proposed to be critical in the development of various vision-threatening diseases [4], such as diabetic retinopathy, AMD, and uveitis. Ang II, the main effector molecule of RAS, contributes to the inflammatory process by increasing the release of pro-inflammatory cytokines, chemokines, and cell adhesion molecules, growth factors, and reactive oxygen species through the AT1R [40]. There is a direct evidence that abnormal production of Ang II in RPE would lead to retinal dysfunction and breakdown of blood-retinal barrier [41]. Ang II was capable of weakening the tight junction of blood-retinal barrier and increasing invasion of the choroidal endothelial cells [42]. On the other hand, AT1R blockers and ACE inhibitors have been shown to decrease the expression of inflammatory cytokines and suppress ocular inflammation [43] as well as vascular inflammation [44]. Recent studies have shown that ACE2 acts as a counter-regulator of ACE in modulating the production of Ang-(1-7) [8, 9]. The ACE2/Ang-(1-7)/Mas axis negatively regulates leukocyte migration and cytokine release and consequently inhibits inflammatory responses [45]. Overexpression of ACE2 reversed the imbalance of ACE2/ACE, reduced Ang II but increased Ang-(1-7) levels, suppressed secretion of IL-1β and tumor necrosis factor (TNF)-α in LPS-stimulated PMVECs [11]. Moreover, ACE2 deletion and Mas receptor inhibition exhibited parallel deleterious effects on LPS-induced damage and cytokine secretion by offsetting the protective effect of ACE2 overexpression [11]. Thus, we proposed that activating ACE2 could exert protection against LPS-induced ocular inflammation.

In line with the previous study, we showed that ACE2 overexpression mediated by DIZE resulted in significant decrease of Ang II and AT1R, whereas Ang-(1-7) was dramatically increased by DIZE. Furthermore, ACE2-siRNA and A779 reversed these effects. Nevertheless, A779 did not affect the expression of AT1R after DIZE treatment in LPS group. A possible explanation is A779 is the antagonist of Ang-(1-7). It blocks the ACE2/Ang-(1-7)/Mas axis, but cannot reduce the expression of ACE2. ACE2 is capable to convert Ang II to Ang-(1-7), thus decrease Ang II and ultimately reduce the expression of AT1R. However, the mRNA expression of AT2R was not affected by any of the treatments. There might be two possibilities. On the one hand, there are large amount of AT2R expressed in the embryonic tissue, but the expression is less in the adult tissues. Either the LPS stimulation will not cause dramatically changes of AT2R expression, or it may not be involved in the LPS-induced response in RPE cells. On the other hand, there is evidence showing when AT1R is blocked, circulating Ang II binds, and activates AT2R only [46]. When the cells are exposed to LPS, Ang II is increased and binds to AT1R, thus the AT2R will not be bound or activated. It is in accordance with our results that the mRNA expression of AT2R was comparable in cells treated with LPS and in untreated cells. In cells treated with DIZE followed by LPS, ACE2 was increased and it could reduce the expression of Ang II, so AT2R was not activated.

DIZE modulates the RAS in a positive manner. It reduces inflammatory cytokines and improves the function of angiogenic progenitor cell in a pulmonary hypertension model [16]. More recently, it was confirmed that DIZE reduced infiltration of inflammatory cells and attenuated ocular inflammatory responses in rodent models of EIU [14, 17]. The protective effect of DIZE was believed to be mediated by activating ACE2. Our study confirmed that the expression of ACE2 increased after DIZE treatment.

We found that DIZE not only significantly augmented the expression of ACE2 in the hRPE and ARPE-19 cells, but also decreased the production of IL-6, IL-8, and MCP-1 when stimulated with LPS. We showed DIZE exerted beneficial effect by activating the expression of ACE2, subsequently alleviated inflammatory response through generating Ang-(1-7) and/or degrading Ang II. Furthermore, ACE2-siRNA and an Ang-(1-7) antagonist A779 offset the protective effect of DIZE. This effect was attributed to the suppression of ACE2/Ang-(1-7)/Mas axis. Therefore, the protective effect of DIZE on LPS-induced inflammation in hRPE and ARPE-19 cells was regulated by activating the ACE2/Ang-(1-7)/Mas axis.

The RPE play an essential role in maintaining normal visual function, and it can be involved in many ocular inflammatory conditions. In response to pro-inflammatory stimuli, RPE are capable of producing a variety of cytokines, which are crucial to ocular inflammation [47]. Our study demonstrated for the first time that enhancing ACE2 exerts anti-inflammatory role in RPE cells. However, there are also many other types of cell are activated during ocular inflammation, such as microglia and Müller cells. Our previous study provided direct evidence that activating ACE2 by DIZE significantly decreased the number of CD45^+^ macrophages in endotoxin-induced uveitis mice [14]. AAV-mediated intravitreal delivery of ACE2 conferred protection against diabetic retinopathy by reducing the retinal CD11b^+^ microglia cell [12]. These data indicated that enhancing ACE2 may be a potential therapeutic target for various ocular inflammatory diseases which not only affect RPE, but also affect other retinal immune cells.

Moreover, in response to inflammatory stimuli, inflammatory response is orchestrated by initiating a series of intracellular signaling cascades and ultimately leads to release of pro-inflammatory cytokines. Prominent cascades among these intracellular signaling pathways include MAPK and NF-κB pathways. LPS initiated the production of pro-inflammatory cytokines by activating the MAPK and NF-κB cascades [48]. In addition, treatment with recombinant ACE2 ameliorated LPS-induced cellular apoptosis and inflammation. A possible underlying mechanism that ACE2 exerts its anti-inflammatory effect is by blocking the activation of p38MAPK and JNK [11]. It has been shown that DIZE dramatically suppressed the production of pro-inflammatory cytokines after LPS treatment by downregulating the phosphorylation of MAPKs (p38MAPK, JNK, ERK), STATs, and NF-κB, which were the key signaling molecules and transcription factors involved in the production of pro-inflammatory cytokines in immune cells [33].

Although activation of the MAPK pathway plays a key role in inflammatory response, its role in RPE cells underwent LPS stimulation has not been fully elucidated. In consistent with the previous studies in other cellular systems [39], we found that the phosphorylation levels of p38MAPK, ERK1/2, and JNK were enhanced in ARPE-19 cells stimulated with LPS. Furthermore, specific inhibitors of p38MAPK, ERK1/2, and JNK decreased the production of inflammatory cytokines. Therefore, all of the three MAPK family members were involved in the LPS-induced inflammatory response in RPE cells. We also showed that DIZE remarkably decreased the phosphorylation of p38MAPK, JNK, and ERK1/2 in response to LPS, whereas ACE2-siRNA and A779 abolished the protective effect of DIZE. Thus, we suggested that DIZE protected against LPS-induced inflammation through the ACE2/Ang-(1-7)/Mas axis by modulating the phosphorylation of p38MAPK, JNK, and ERK1/2.

The NF-κB pathway is pivotal to the inflammatory response. NF-κB normally resides in the cytoplasm as an inactive form complex with an inhibitor namely transcription inhibition factor-κB (IκB). External stimuli activate NF-κB transcription, lead to the phosphorylation of IκB-α, and increase the degradation of IκB by proteosome [49]. NF-κB is activated and translocated into the nucleus to upregulate the transcription of several inflammatory genes. Many in vitro and in vivo studies demonstrated that activation of NF-κB is the key link in regulating the LPS-induced inflammatory response [39, 50]. Inhibiting NF-κB signaling reduced the pro-inflammatory cytokines production in LPS-induced uveitis and in RPE cells [51]. Here, we confirmed that LPS increased the activation of NF-κB in RPE cells, and the specific inhibitor of NF-κB significantly decreased the pro-inflammatory cytokines generation. Our results proved that the NF-κB signaling was involved in LPS-induced inflammatory response in ARPE-19 cells. More interestingly, we found that DIZE remarkably suppressed the activation of NF-κB induced by LPS. In addition, ACE2-siRNA and pre-treatment of A779 abrogated the inhibition effect of DIZE on NF-κB pathway, suggesting that the beneficial effect of DIZE on LPS-induced inflammation was through modulating the ACE2/Ang-(1-7)/Mas axis by down-regulating the activation of NF-κB pathway.

## Conclusions

In conclusion, we demonstrate that by activating the protective axis of RAS, ACE2/Ang-(1-7)/Mas, DIZE exerts protective effects on LPS-induced inflammatory response in hRPE and ARPE-19 cells. Our results strongly suggest that the anti-inflammatory effect of DIZE is mediated by inhibiting the MAPK and NF-κB pathways. Upregulation of the ACE2/Ang-(1-7)/Mas axis is beneficial in the LPS-induced inflammation. It may shed light on finding a novel promising therapeutic strategy against ocular inflammation.

## Abbreviations

ACE: angiotensin-converting enzyme
AMD: age-related macular disease
Ang II: angiotensin II
Ang-(1-7): angiotensin-(1-7)
AT1R: angiotensin II type 1 receptor
AT2R: angiotensin II type 2 receptor
CNV: choroidal neovascularization
DIZE: diminazene aceturate
EIU: endotoxin-induced uveitis
ERK1/2: signal-regulated kinase 1/2
hRPE: human primary retinal pigment epithelium
IL-6: interleukin-6
IκB: inhibition factor-κB
JNK: c-Jun N-terminal kinase
LPS: lipopolysaccharide
MAPK: mitogen-activated protein kinase
MCP-1: monocyte chemoattractant protein-1
NF–κB: nuclear factor-κB
RAS: renin-angiotensin system
RPE: retinal pigment epithelium
